# Supplementary material for: Engineering polar nanoclusters for enhanced microwave tunability in ferroelectric thin films
Source: Nat Commun. 2025 Oct 31;16:9643. doi: 10.1038/s41467-025-64642-1 (PMC12579223; doi:10.1038/s41467-025-64642-1)
Supplement: Supplementary file 1 — Supplementary Information [file 41467_2025_64642_MOESM1_ESM.pdf]

# Supplementary Materials for

## Engineering polar nanoclusters for enhanced microwave tunability in ferroelectric thin films

Hanchi Ruan[1], Hangfeng Zhang[1], Vladimir Roddatis[2], Subhajit Pal[3], Joe Briscoe[3], Theo Graves Saunders[1], Xuyao Tang[3], Haixue Yan[3*], Yang Hao[1*]

* Corresponding author.

Email: *H. Yan:* h.x.yan@qmul.ac.uk; Y. Hao: y.hao@qmul.ac.uk

**The PDF file includes:**

Figs. S1 to S11

Tables S1 to S4

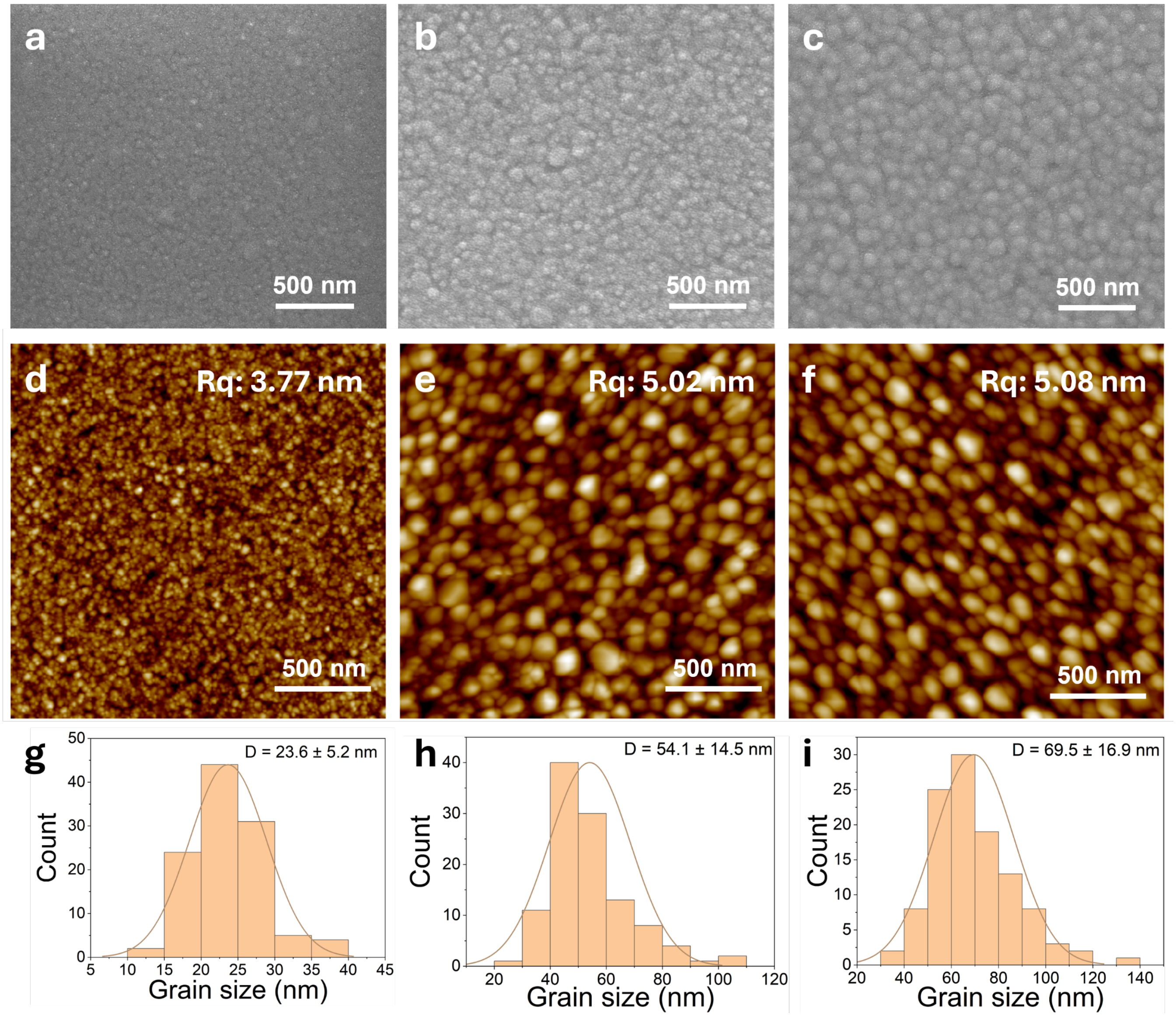


**Supplementary Fig. 1** The surface SEM images of (**a**) BTS800 thin film, (**b**) BTS850 thin film, and (**c**) BTS900 thin film. AFM surface topographies of (**d**) BTS800, (**e**) BTS850 and (**f**) BTS900 with the surface roughness (Rq) inset. Grain size distribution extracted from AFM images for (**g**) BTS800, (**h**) BTS850 and (**i**) BTS900.

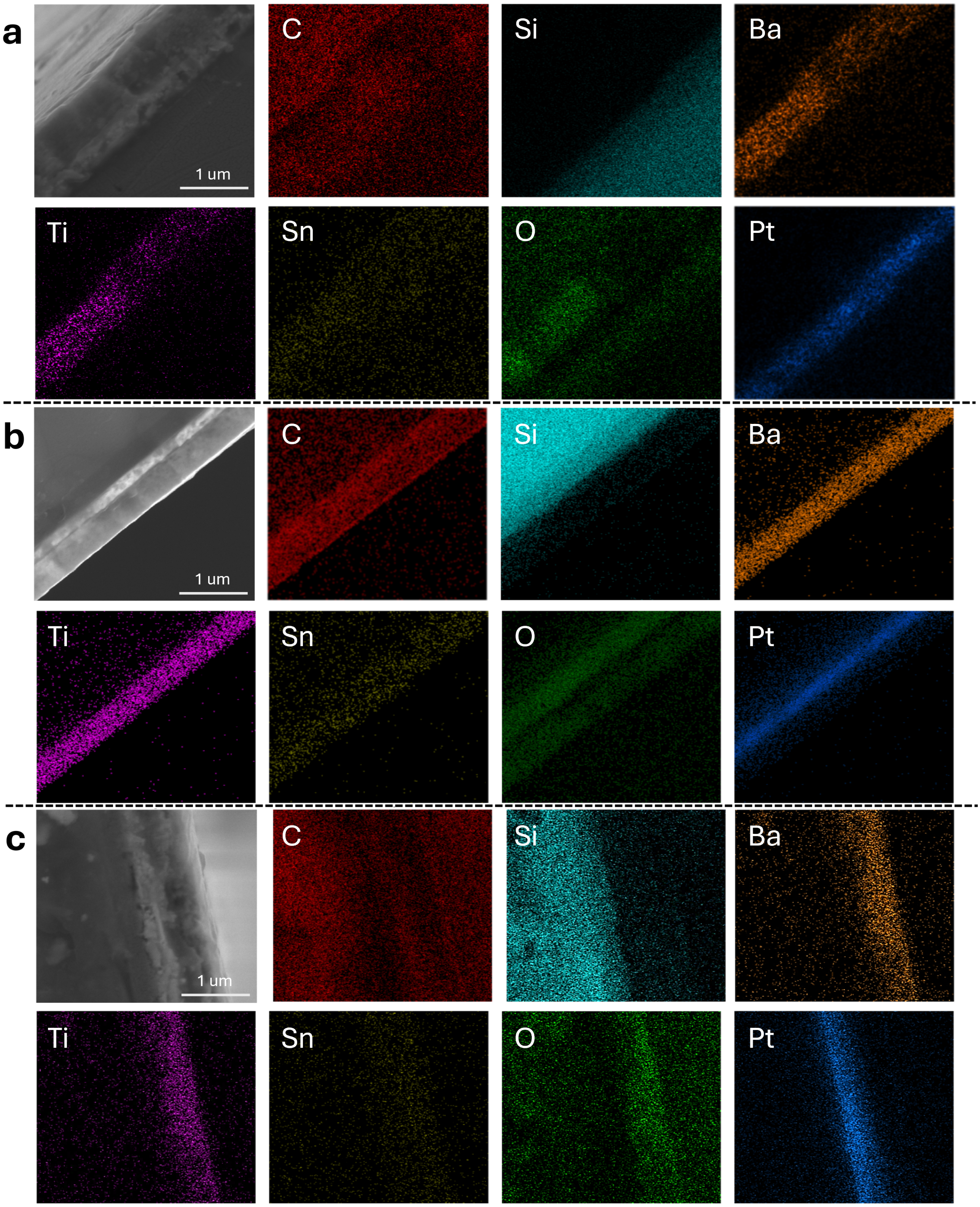


**Supplementary Fig. 2** SEM images of cross-section of **a** BTS800, **b** BTS850 and **c** BTS900, alongside their EDX elemental mappings.

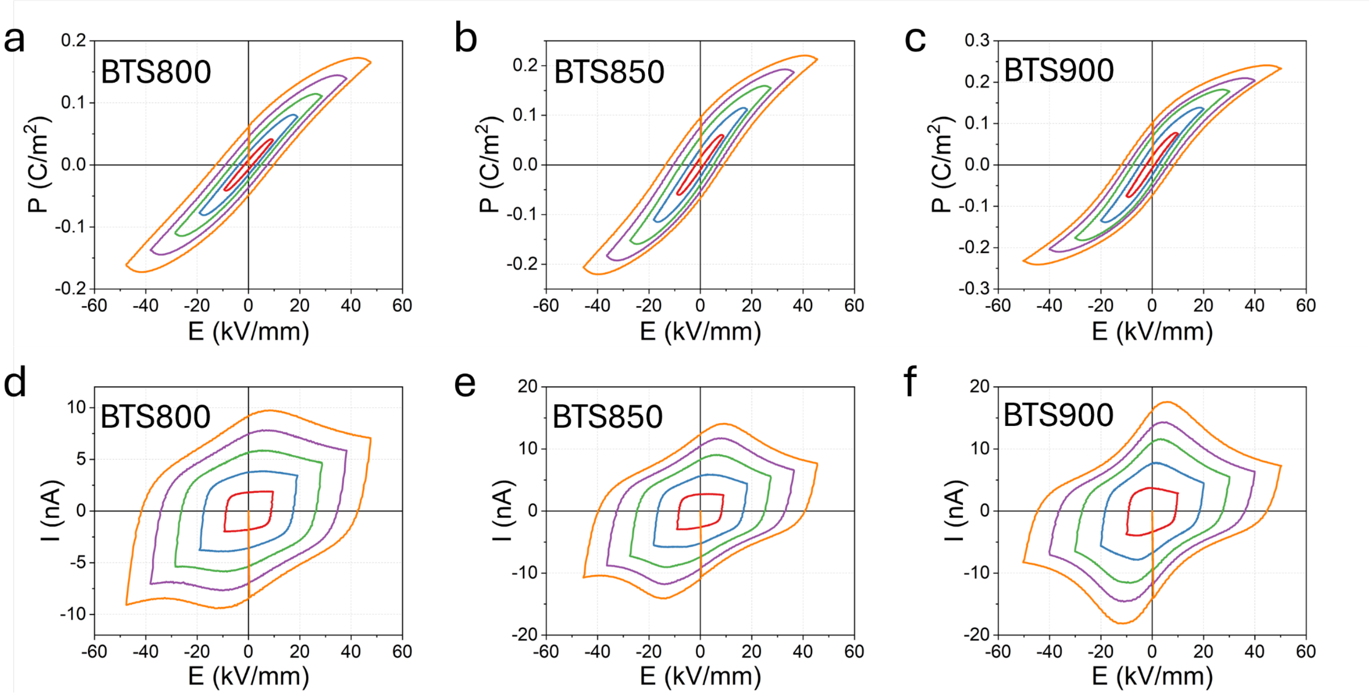


**Supplementary Fig. 3 a-c** *P-E* loop and **d-f** *I-E* loop of the BTS thin film measured under applied AC voltages of 5 V, 10 V, 15 V, 20 V, and 25 V, which were all measured at 100 Hz and at room temperature 20 °C. The different applied electric fields were obtained by calculating the ratio of the applied voltage to the film thickness.

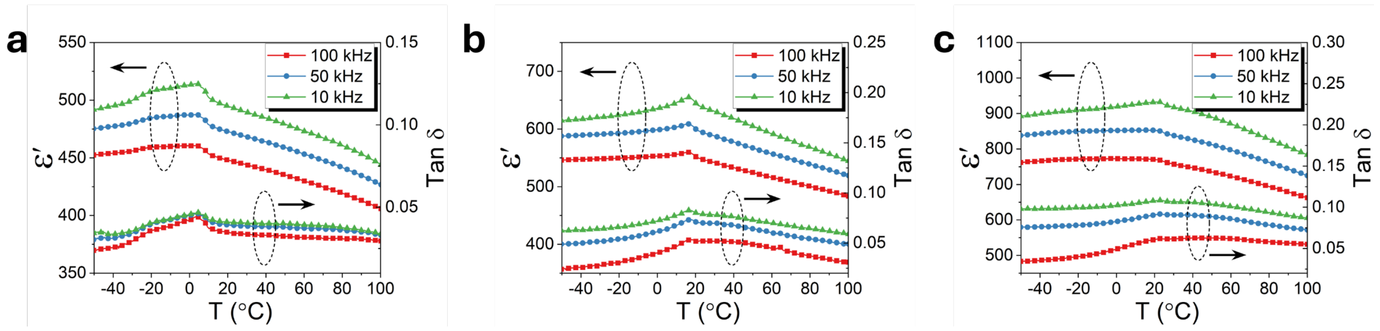


**Supplementary Fig. 4** Temperature-dependent dielectric permittivity and loss tangent of **a** BTS800 thin film; **b** BTS850 thin film; and **c** BTS900 thin film at selected frequencies of 10 kHz, 50 kHz and 100 kHz.

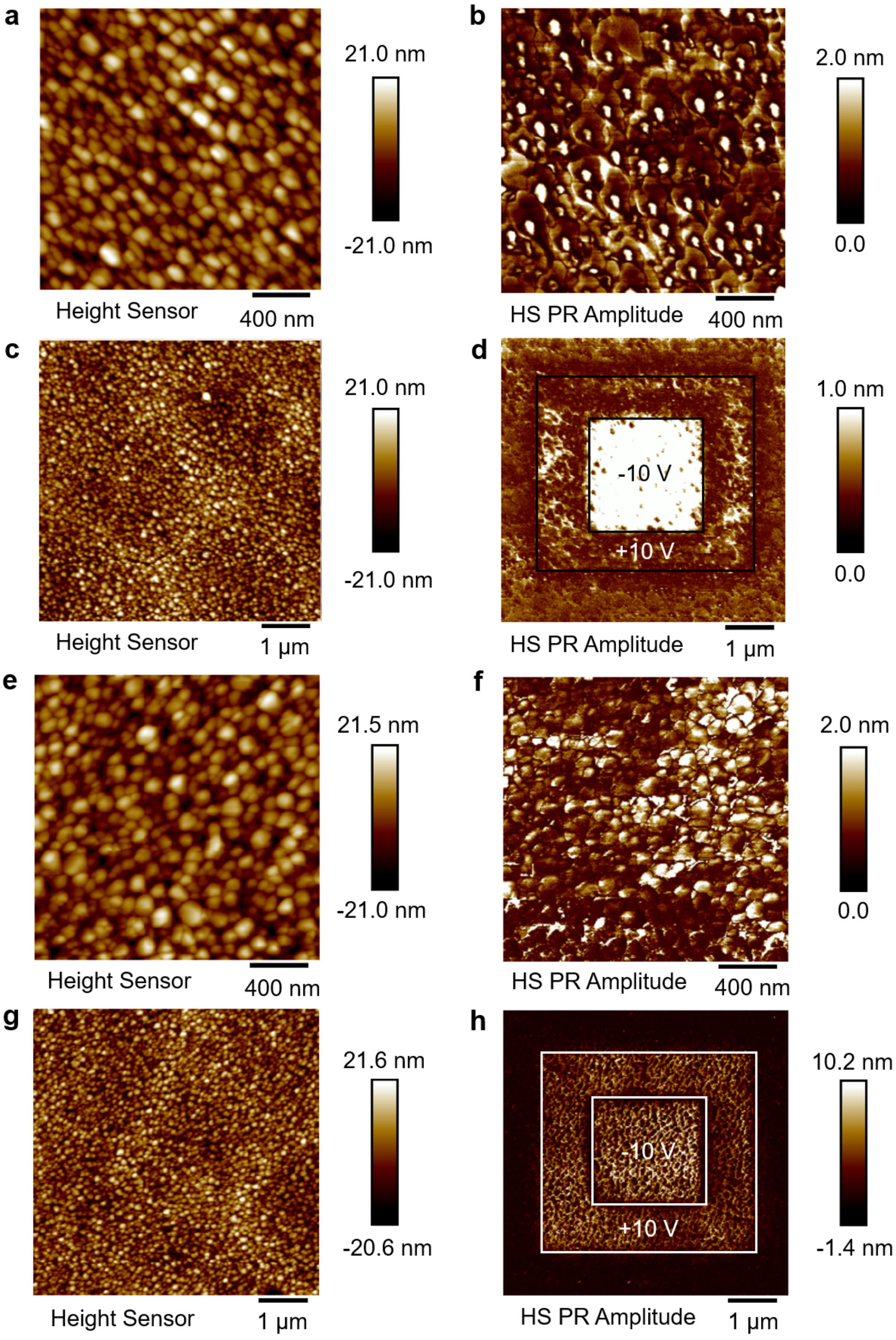


**Supplementary Fig. 5** PFM images of the BTS850 thin film. **a** topography image and **b** amplitude image measured at 14 °C and reading at applied AC of 3 V (**a** and **b** were measured together with the phase image as shown in Fig. 3a). **c** topography image and **d** amplitude image (with opposite polarization direction after applying a ±10 V DC bias to the tip) measured at 14 °C and reading at applied AC of 2 V (**d** was measured together with the phase-contrast PFM image as shown in Fig. 3b). **e** topography image and **f** amplitude image measured at 21 °C (**e** and **f** were measured together with the phase image as shown in Fig. 3d). **g** topography image and **h** amplitude image associated with the phase image of Fig. 3e.

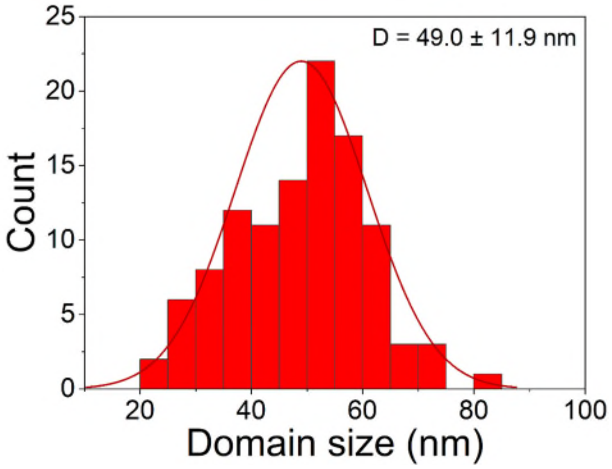


**Supplementary Fig. 6** The domain size distribution of the BTS850 thin film. The average domain size and its standard deviation based on its count are shown as the D value.

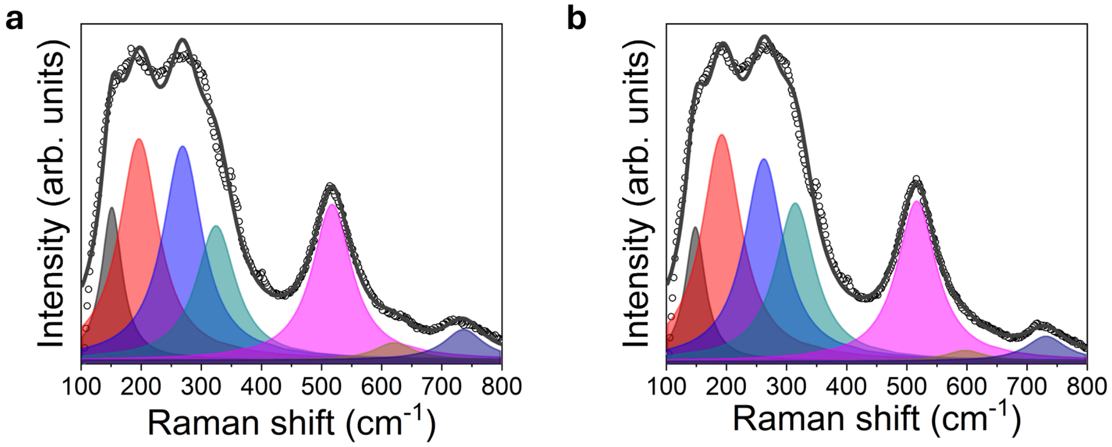


**Supplementary Fig. 7** Fitted Raman spectra for **a** BTS800 and **b** BTS900 thin films.

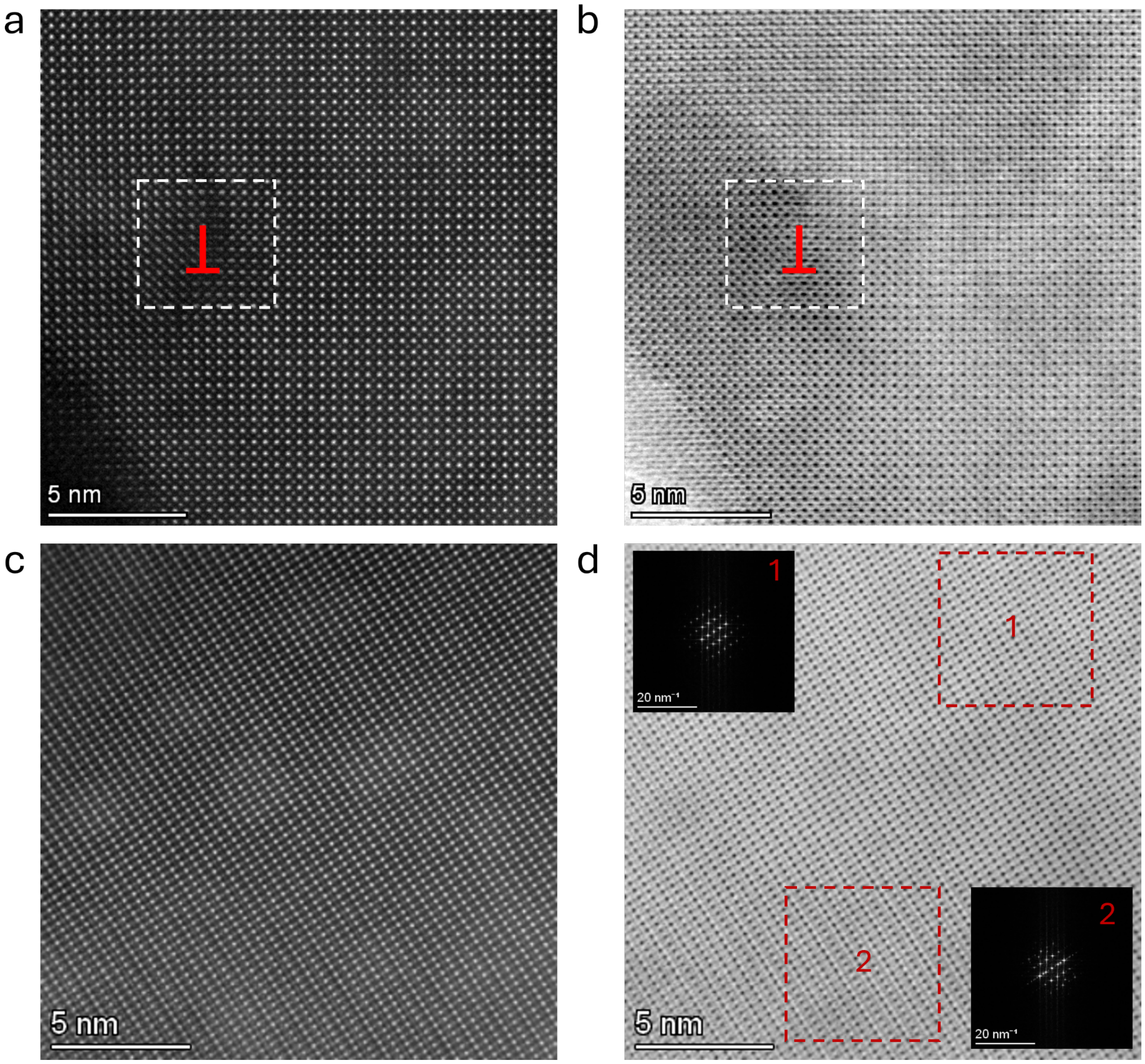


**Supplementary Fig. 8** High-resolution HAADF (a, c) and ADF (b, d) images of BTS850 reveal a nanoscale region with distinct contrast (marked by ⊥) embedded in periodic matrix. (d) Two distinct regions (label 1 and 2), with corresponding SEAD (inset) patterns showing the extra diffraction spots in region 2.

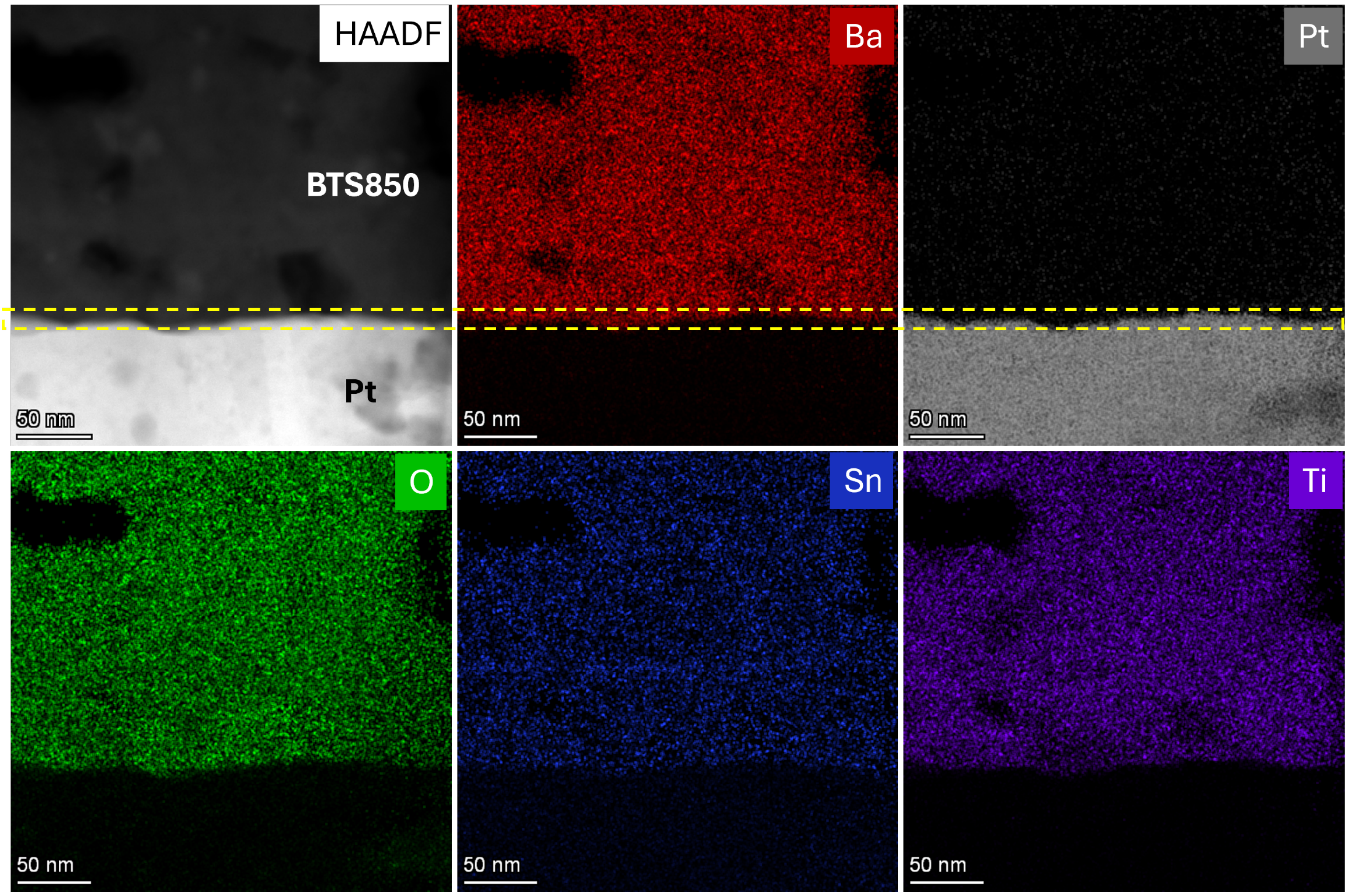


**Supplementary Fig. 9** HAADF image and corresponding EDX elemental maps showing the elemental distribution of Ba, Ti, Sn, O, and Pt across the BTS850/Pt interface.

**a**

**b**

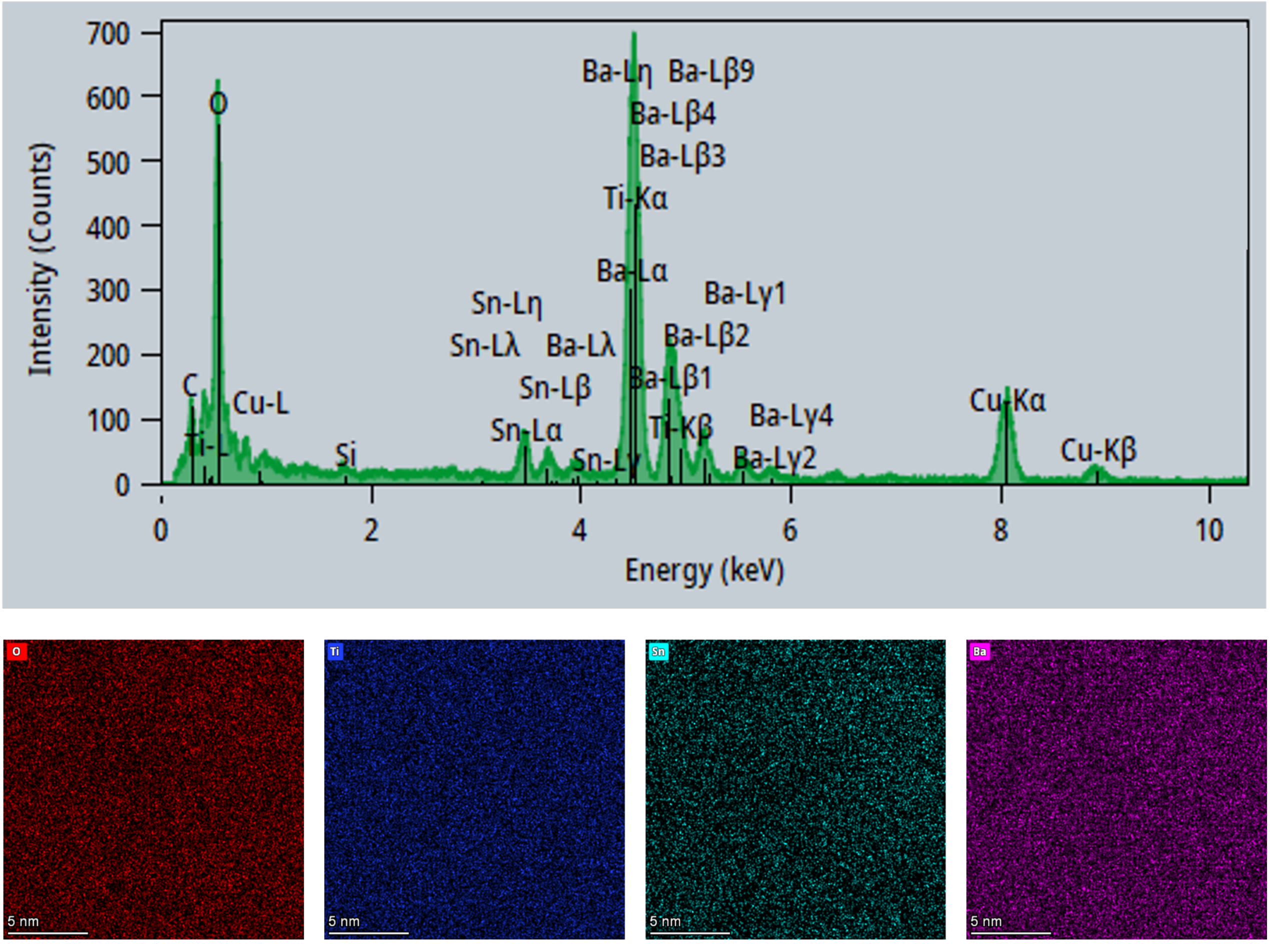


**Supplementary Fig. 10 a** Integrated EDX spectrum and **b** uniform corresponding elemental maps of O, Ti, Sn and Ba.

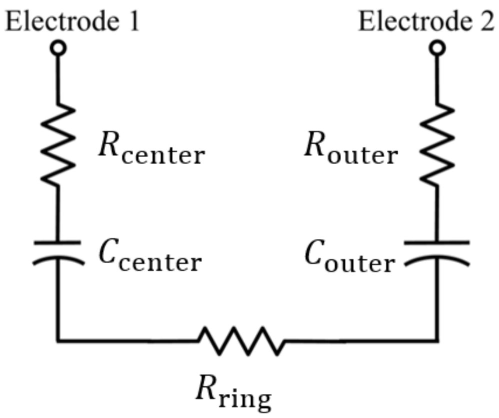


**Supplementary Fig. 11** Simplified equivalent circuit of the parallel-plate varactor. Grain interior and grain-boundary regions are represented by ideal capacitors ($C_{center}$, $C_{outer}$) and resistors ($R_{center}$, $R_{outer}$) linked via $R_{ring}$. Polydisperse effects will appear as deviations from a perfect 90° capacitive phase, which can be captured by modelling $C_{center}$ and $C_{outer}$ as constant-phase elements (CPEs) in a more detailed impedance analysis. This schematic is intended to show the topological mapping between microstructure and circuit.

**Supplementary Table 1** Refined crystal parameters of studied compositions with standard error in the parentheses.

| **Chemical formula** | **$BaTi_{0.85}Sn_{0.15}O_3$** | | |
|---|---|---|---|
| **Sintering temperature** | 800 °C | 850 °C | 900 °C |
| **Space group** | *Pm*-3*m* | *Pm*-3*m* | *Pm*-3*m* |
| **Unit cell dimensions (Å)** | *a* = 4.0225(2)<br>*b* = 4.0225(2)<br>*c* = 4.0225(2) | *a* = 4.0238(2)<br>*b* = 4.0238(2)<br>*c* = 4.0238(2) | *a* = 4.0260(4)<br>*b* = 4.0260(4)<br>*c* = 4.0260(4) |
| **Volume (Å$^3$)** | 65.08(1) | 65.15(1) | 65.25(2) |
| **Z** | 1 | 1 | 1 |
| **$D_{calc}$ (g cm$^{-3}$)** | 6.221 | 6.215 | 6.205 |
| **R-factors** | $R_{wp}$ = 0.0862<br>$R_p$ = 0.0584<br>$R_F^2$ = 0.2386<br>$\chi^2$ = 11.02 | $R_{wp}$ = 0.0868<br>$R_p$ = 0.0615<br>$R_F^2$ = 0.1570<br>$\chi^2$ = 11.02 | $R_{wp}$ = 0.0931<br>$R_p$ = 0.0670<br>$R_F^2$ = 0.1564<br>$\chi^2$ = 15.50 |
| **No. of variables** | 35 | 37 | 35 |
| **No. of profile points** | 3490 | 3490 | 3490 |

**Supplementary Table 2** Elemental composition of BTS850 thin film from area b in Fig.4a

| Z | Element | Family | Atomic Fraction (%) | Atomic Error (%) | Mass Fraction (%) | Mass Error (%) | Fit Error (%) |
|---|---|---|---|---|---|---|---|
| 6 | C | K | 14.57 | 1.34 | 3.98 | 0.27 | 0.70 |
| 8 | O | K | 41.41 | 5.12 | 15.09 | 2.72 | 1.33 |
| 14 | Si | K | 0.60 | 0.13 | 0.38 | 0.08 | 3.52 |
| 22 | Ti | K | 20.30 | 3.23 | 22.13 | 3.17 | 0.24 |
| 29 | Cu | K | 7.56 | 1.22 | 10.94 | 1.57 | 0.31 |
| 50 | Sn | L | 2.70 | 0.38 | 7.29 | 0.89 | 0.46 |
| 56 | Ba | L | 12.85 | 1.69 | 40.18 | 3.33 | 0.27 |

**Supplementary Table 3** Elemental composition of BTS850 thin film from area c in Fig.4a

| Z | Element | Family | Atomic Fraction (%) | Atomic Error (%) | Mass Fraction (%) | Mass Error (%) | Fit Error (%) |
|---|---|---|---|---|---|---|---|
| 6 | C | K | 15.55 | 1.40 | 4.26 | 0.29 | 1.34 |
| 8 | O | K | 39.89 | 5.06 | 14.54 | 2.64 | 0.78 |
| 14 | Si | K | 0.58 | 0.12 | 0.37 | 0.08 | 4.02 |
| 22 | Ti | K | 20.99 | 3.29 | 22.90 | 3.25 | 0.39 |
| 29 | Cu | K | 7.76 | 1.24 | 11.24 | 1.61 | 0.24 |
| 50 | Sn | L | 2.26 | 0.32 | 6.12 | 0.75 | 0.58 |
| 56 | Ba | L | 12.96 | 1.68 | 40.57 | 3.36 | 0.24 |

**Supplementary Table 4** Comparison of microwave dielectric tunability, loss, and figure of merit (FoM) for tunable dielectric films synthesized by different methods.

| Composition | Synthesis method | Frequency (GHz) | Electric field (kV/cm) | Tunability (%) | Loss | FoM | Reference |
|---|---|---|---|---|---|---|---|
| $Sr_6BaTi_6O_{19}$ | MBE | 10 | 400 | 46 | 0.018 | 25 | 1 |
| $Ba_{0.5}Sr_{0.5}TiO_3$ | PLD | 10 | 67 | 20 | 0.05 | 20 | 2 |
| $Pb_{0.35}Sr_{0.65}TiO_3$ | PLD | 10 | 40 | 35 | 0.1 | 3.5 | 3 |
| $AgTa_{0.1}Nb_{0.9}O_3$ | SP | 4 | 10 | 5 | 0.1 | 0.5 | 4 |
| $Ba_{0.6}Sr_{0.4}TiO_3$ | PLD | 8 | 200 | 20 | 0.01 | 20 | 5 |
| $Pb_{0.4}Sr_{0.6}Ti_{0.97}Mn_{0.03}O_3$ | Sol-gel | 10 | 240 | 50 | 0.05 | 10 | 6 |
| $BaSn_{0.15}Ti_{0.85}O_3$ | Sol-gel | 6 | 273 | 74 | 0.020 | 37 | This work |

Note: MBE (molecular-beam epitaxy), PLD (pulsed laser deposition) and SP (screen printing)

Reference,


1. Dawley, N. M. *et al.* Targeted chemical pressure yields tuneable millimetre-wave dielectric. *Nat. Mater.* **19**, 176–181 (2020).
2. Chang, W. *et al.* The effect of annealing on the microwave properties of Ba0.5Sr0.5TiO3 thin films. *Appl. Phys. Lett.* **74**, 1033–1035 (1999).
3. Liu, S. W. *et al.* Ferroelectric (Pb,Sr)TiO3 epitaxial thin films on (001) MgO for room temperature high-frequency tunable microwave elements. *Appl. Phys. Lett.* **87**, 142905 (2005).
4. Zimmermann, F., Menesklou, W. & Ivers-Tiffée, E. Investigation of Ag(Ta,Nb)O3as tunable microwave dielectric. *J. Eur. Ceram. Soc.* **24**, 1811–1814 (2004).
5. Chang, W., Kirchoefer, S. W., Pond, J. M., Horwitz, J. S. & Sengupta, L. Strain-relieved Ba 0.6Sr 0.4TiO 3 thin films for tunable microwave applications. *J. Appl. Phys.* **92**, 1528–1535 (2002).
6. Fragkiadakis, C., Lüker, A., Wright, R. V., Floyd, L. & Kirby, P. B. Growth and high frequency characterization of Mn doped sol-gel Pb xSr1-xTiO3 for frequency agile applications. *J. Appl. Phys.* **105**, (2009).
